# Cannabidiol Encapsulation in Polymeric Hydrogels and Its Controlled Release: A Review

**DOI:** 10.3390/gels11100815

**Published:** 2025-10-11

**Authors:** Víctor M. Ovando-Medina, Carlos A. García-Martínez, Lorena Farias-Cepeda, Iveth D. Antonio-Carmona, Andrés Dector, Juan M. Olivares-Ramírez, Alondra Anahí Ortiz-Verdin, Hugo Martínez-Gutiérrez, Erika Nohemi Rivas Martínez

**Affiliations:** 1 Ingeniería Química, UAMRA—Universidad Autónoma de San Luis Potosí, Carretera a Cedral KM 5+600, San José de las Trojes, Matehuala 78700, San Luis Potosí, Mexico; 2 Departamento de Ingeniería Química, Facultad de Ciencias Químicas, Universidad Autónoma de Coahuila, Blvd. V. Carranza e Ing. José Cárdenas V. S/N, Saltillo 25280, Coahuila, Mexico; c-garciamartinez@uadec.edu.mx; 3 Departamento de Botánica, Universidad Autónoma Agraria Antonio Narro, Calzada Antonio Narro 1923, Buenavista, Saltillo 25315, Coahuila, Mexico; dalila_antonio@yahoo.com (I.D.A.-C.); erika_rivas257@outlook.com (E.N.R.M.); 4 Secretaría de Ciencia, Humanidades, Tecnología e Innovación (SECIHTI), Universidad Tecnológica de San Juan del Rio, San Juan del Rio 76800, Querétaro, Mexico; andres_dector@live.com; 5 Division de Quimica y Energia y Desarrollo Sostenible, Universidad Tecnológica de San Juan del Rio, Av. La Palma No 125 Vista Hermosa, San Juan del Rio 76800, Querétaro, Mexico; jmolivar01@yahoo.com; 6 Industrial Technologies Division, Universidad Politecnica de Queretaro, El Marques 76240, Querétaro, Mexico; alondra.ortiz@upq.mx; 7 Centro de Nanociencias y Micro y Nanotecnologías, Instituto Politécnico Nacional (IPN), Ciudad de México 07700, Mexico; humartinez@ipn.mx

**Keywords:** hydrogels, CBD, cannabidiol, encapsulation, polymeric hydrogels

## Abstract

Cannabidiol (CBD) and its derivatives show interesting therapeutic potential, including antioxidant, anti-inflammatory, and anticancer properties; however, their clinical translation remains a complex task due to physicochemical restrictions such as low water solubility, high lipophilicity, and instability under light, oxygen, and high temperatures. Polymeric encapsulation has emerged as a promising strategy to overcome these challenges, offering protection against environmental degradation, improved bioavailability, and controlled release. Natural and synthetic polymers, both biocompatible and biodegradable, provide versatile matrices for CBD delivery, enabling nanoparticle formation, targeted transport, and enhanced pharmacokinetics. This review highlights the structural characteristics of CBD, its interaction mechanisms with polymeric matrices such as hydrogels, electrospun nanofibers, biodegradable microparticles, thin films, and lipid-polymer hybrid systems, and the principal encapsulation techniques, such as emulsion solvent evaporation, electrospinning, and supercritical fluid technologies, that facilitate stability and scalability. Furthermore, material characterization approaches, including microscopy, thermal, and degradation analyses, are discussed as tools for optimizing encapsulation systems. While notable advances have been made, key challenges remain in achieving reproducible large-scale production, ensuring regulatory compliance, and designing smart polymeric carriers personalized for specific therapeutic contexts. By addressing these gaps, polymer-based encapsulation may unlock new opportunities for CBD in pharmaceutical, nutraceutical, and therapeutic applications, providing a guide for future innovation and translation into effective patient-centered products.

## 1. Introduction

Cannabis has been used for centuries due to its medicinal properties, but the negative applications, such as its recreational use, have led to the prohibition of these compounds [1]. The study of cannabinoid delivery led to the discovery of the endogenous cannabinoid system and its important role in other major systems such as the immune and central nervous systems [2]. Cannabis is the main source of cannabinoids, which are terpenophenolic compounds that modulate neurotransmitters in the brain via cannabinoid receptors [3]. CBD and Δ^9^-Tetrahydrocannabinol (THC) are among the main cannabinoids of the cannabis plant (Cannabis sativa, Cannabis indica, and hemp); hemp is the primary source of CBD, as studies have shown it produces higher amounts of this bioactive compound. CBD is classified as a phytocannabinoid because it is naturally produced in cannabis plants [4].

The principal difference between CBD and other phytocannabinoids is that it does not generate addiction, as it is non-psychotropic. Therefore, it has been the subject of study in the pharmaceutical field in recent years and has been shown to contain antioxidant, anti-inflammatory, and anticancer properties, with applications in neurological, cardiac, gastrointestinal, and cancerous diseases [5]. Currently, Epidiolex^®^ is the only cannabidiol-based medicine approved by both the FDA and the EMA for the treatment of severe epileptic syndromes, while other CBD-containing formulations are commercialized as dietary supplements or cosmetics without full therapeutic authorization.

**Figure 1:**
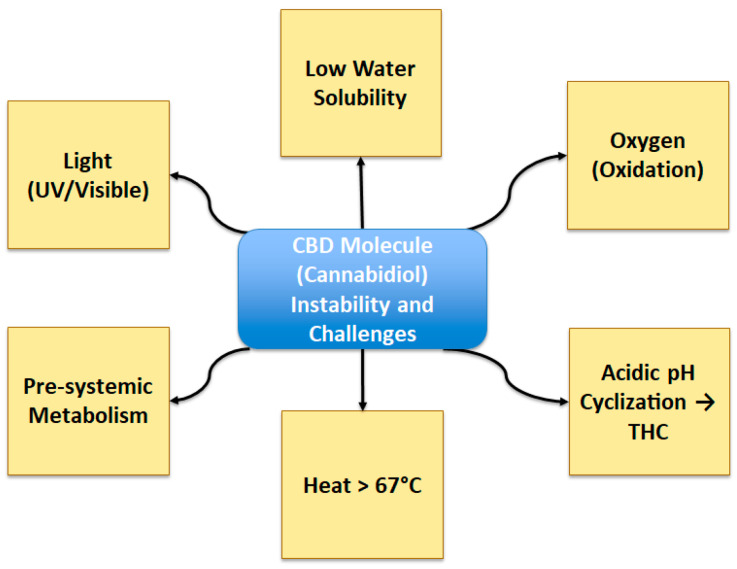
Schematic overview of CBD instability and challenges.

Many disadvantages of cannabinoid compounds such as CBD include their high hydrophobicity, as they are highly lipophilic (Figure 1). Another disadvantage is their sensitivity to environmental conditions such as temperature, light, and oxygen [3]. At room temperature, CBD is a crystalline solid, but when exposed to temperatures above 67 °C or light, it undergoes the formation of mono- and dimeric hydroquinones and degradation. In the presence of oxygen, the structure of CBD is oxidized, generating quinones. Under strongly acidic laboratory conditions, CBD may cyclize to Δ^9^-THC or other cannabinoids; however, this transformation is minimal under physiological conditions and is not considered relevant in vivo. This behavior has been mainly observed under experimental conditions designed to simulate gastric acid exposure, rather than under normal biological environments [6].

Due to these conditions, ensuring the stability and delivery of pharmaceutical products containing CBD generates a high level of difficulty. Another disadvantage of using CBD as a biomedical treatment is its low water solubility (0.7–10 µg/mL [7]) and susceptibility to presystemic metabolism, meaning that breakdown can occur before achieving an effect at the target site in the body. For this reason, various research efforts focus on methods of release and administration of the compound [8]. An excellent alternative to ensure stability and enhance delivery is the encapsulation of CBD within polymeric or organic matrices. Encapsulation not only protects bioactive compounds from degradation but also improves their bioavailability and bioaccessibility, making it particularly useful in pharmaceutical and dietary supplement development [9].

Encapsulation involves covering an organic or inorganic material with another material to protect it from environmental conditions. Polymeric materials are an excellent alternative for encapsulating bioactive compounds. Their use in pharmaceutical, cosmetic, and food products is supported by multiple rationales; one of the most important is cost-effectiveness, as the production of polymeric materials is often less expensive while simultaneously offering high stability and biocompatibility [10]. Another reason for the use of polymeric materials in the encapsulation of bioactive compounds is the possibility of generating polymeric nanoparticles (PNPs). In recent years, significant attention has been given to the production of efficient nanomaterials.

One of the major challenges in drug development is achieving effective delivery across complex biological barriers [11]. Numerous studies have demonstrated that nanoscale drug carriers functionalized with polymer coatings can markedly enhance bioavailability, biocompatibility, safety, and therapeutic performance. Such systems not only improve safety profiles but also reduce toxicity, while simultaneously promoting better absorption, permeability, and prolonged retention time [12]. The modification of polymers can permit interactions with drugs in the biological system, promoting absorption into targeted tissues and efficient transport in the bloodstream. In recent years, the use of FDA-approved polymers has gained prominence as a strategy to develop polymeric materials capable of effectively encapsulating drugs and bioactive compounds, thereby enhancing their stability under environmental conditions and improving delivery within biological systems [12].

In this review, the objective is to present and analyze the encapsulation of bioactive compounds such as CBD and its derivatives using polymeric materials as an efficient strategy to enhance their physicochemical properties, biological performance, and therapeutic potential. This work gathers essential information about these compounds, including their chemical nature, structural characteristics, and current or potential applications as effective pharmacological agents for the treatment of various diseases, as well as their use in therapeutic and psychotherapeutic contexts. Furthermore, we explore the different encapsulation techniques utilizing polymeric carriers, assessing their effectiveness in improving critical aspects such as controlled release in biological systems, enhanced environmental stability, and targeted delivery through structural modifications of the encapsulating matrix. In addition, special attention will be given to both natural and synthetic polymeric materials, highlighting their individual advantages, biocompatibility, and adaptability in the encapsulation of bioactive substances. Their evolution, performance, and growing versatility in pharmaceutical and therapeutic applications will also be discussed, offering a comprehensive perspective on current trends and future directions in this field.

## 2. Physicochemical Properties of CBD and Its Derivatives

Cannabinoids are a diverse class of bioactive compounds that share structural similarities and comparable biological activities with the primary cannabis-derived cannabinoid, tetrahydrocannabinol (THC). However, cannabinoids are classified into three classes: The first class is the endogenous cannabinoids or endocannabinoids; these compounds are produced naturally in the body. Unlike other classes, plant-derived cannabinoids, known as phytocannabinoids, occur exclusively in the cannabis plant and represent the most widely studied and recognized group of cannabinoid compounds. In contrast, synthetic cannabinoids are produced in laboratories as structural and chemical analogues of both endocannabinoids and phytocannabinoids [13].

### 2.1. Molecular Structure, Solubility, and Stability Issues

**Figure 2:**
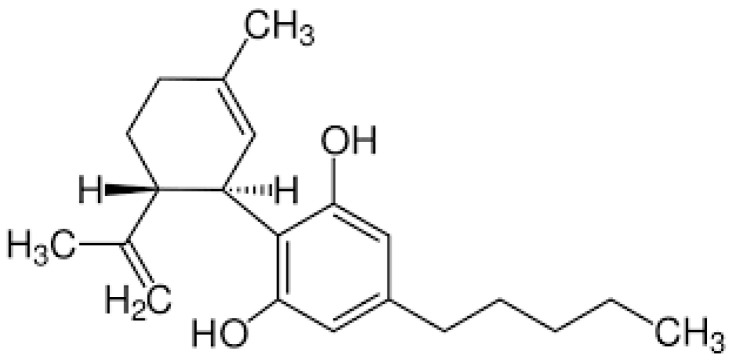
Chemical structure of CBD molecule.

The chemical structure of CBD (Figure 2) is like that of THC. However, CBD is thought to possess a tetrahydrobiphenyl structure, consisting of a bicyclic core that results from the combination of the monoterpene *p*-cymene and the alkylresorcinol compound olivetol [6]. However, CBD with a dibenzo[b,d]pyran structure is derived biosynthetically from cannabigerolic acid (CBGA) through enzymatic cyclization, rather than by direct combination of *p*-cymene and olivetol. Furthermore, structurally, CBD is composed of 21 carbon atoms. Its structure includes a cyclohexane ring, a phenol group, and a pentyl side chain (although natural CBD homologs may also contain methyl, propyl, or butyl side chains). The two rings, which have a trans configuration, are arranged almost perpendicularly to each other (see Figure 2) [6,14]. One consequence of this chemical structure is the compound’s poor water solubility, accompanied by both chemical and metabolic instability. Due to its low solubility and limited oral bioavailability (estimated at around 6 to 20%), the therapeutic application of this compound has been restricted, requiring alternative delivery methods [15]. Additionally, its high instability under environmental conditions leads to rapid degradation.

Stability studies of cannabinoids have shown that these compounds are highly unstable under environmental conditions. Most of the available research has focused on THC, possibly because it is the most widely used cannabinoid. However, studies on CBD have reported similar behavior, highlighting its thermolability, susceptibility to oxidation in the presence of air, instability under acidic conditions, and sensitivity to light (photolability) [16]. The thermal stability of CBD has been reported to be low, with a melting point of approximately 67 °C [17]. Under various unfavorable conditions, the molecular structure of CBD tends to degrade, which can significantly reduce its pharmacological efficacy.

Under ambient storage at 25 °C, protected from light and oxygen, CBD in the form of powder is stable, with less than 7% degradation after 1 year. However, at higher temperatures, degradation accelerates. Studies report between 9 and 11% degradation at 40 °C after 1 year due to thermal oxidation and isomerization reactions. Usually, CBD is stored as a sunflower oil solution due to its good bioavailability. Then, when stored in closed vials at 25 °C and 40 °C, the CBD oil solution degraded by around 40%, and 100% decomposition occurred after 1 year [18]. Light exposure is one of the most critical factors. Under sunlight, CBD suffers photooxidation, leading to the formation of cannabielsoin, with degradation reaching up to 100% exposed to air after only 40 h [19].

In the study by Jaidee et al. [20], the degradation kinetics of cannabidiol (CBD) were studied at different pH and temperature conditions. CBD degradation followed first-order kinetics in aqueous solution and zero-order kinetics in cannabis resin, with the rate strongly dependent on both pH and temperature. The compound was stable between pH 4 and 6, whereas under pH 2 and 70 °C, CBD exhibited rapid degradation, with over 80% loss after 24 h. The authors attributed CBD instability primarily to acid-catalyzed cyclization to Δ^9^-THC and oxidation processes enhanced by thermal stress.

### 2.2. Interaction of CBD with Polymeric Materials

Encapsulation is a key technology for enhancing the stability of natural compounds under environmental conditions, with a wide range of materials available for this purpose. In recent years, polymers have emerged as particularly attractive candidates for the encapsulation and recovery of pharmaceutical products and bioactive compounds. Several studies highlight polymers as promising materials for drug delivery systems, offering advantages in terms of rheological properties, transport efficiency, and controlled release [21] and stability to encapsulate compounds.

While most encapsulation studies employ purified cannabidiol (CBD), it is important to admit that hemp-derived full- and broad-spectrum extracts are widely used in commercial and clinical formulations. These extracts contain additional phytocannabinoids, terpenes, and flavonoids that may contribute to the so-called “entourage effect,” influencing therapeutic efficacy and stability. From a technological point of view, the presence of diverse lipophilic compounds modifies solubility profiles and polymer–drug interactions, often requiring emulsifiers or co-solvents to achieve homogeneous encapsulation. Moreover, such extracts are generally subject to stricter quality control and labeling requirements, and their legal classification varies by jurisdiction, frequently falling under food supplements or cosmetic categories rather than pharmaceutical approval frameworks. Consequently, while this review focuses primarily on pure CBD systems for clarity and comparability, the encapsulation of full-spectrum hemp extracts represents a relevant and growing research direction with unique formulation and regulatory challenges.

There are various studies on the encapsulation of CBD and other cannabinoid compounds; however, research specifically focused on the encapsulation of these compounds using polymeric materials remains limited. One of the key features of encapsulation techniques involving polymeric matrices is the ability to obtain particles at the nanoscale. According to recent studies, CBD nanoparticles encapsulated in polymeric matrices can be successfully obtained [16], offering an efficient size range that enhances releases within biological systems, thereby improving cellular absorption, permeability, and overall bioavailability of the compound [22].

A wide range of polymers has been explored for encapsulation, including natural polymers such as chitosan, alginate, and gelatin, and synthetic polymers such as poly(lactic-co-glycolic acid) (PLGA), polycaprolactone (PCL), and polyethylene glycol (PEG). Many of these are FDA-approved, which underscores their potential for clinical translation. The choice of polymer strongly influences release kinetics, stability, and compatibility.

One of the advantages associated with polymer-based encapsulation is that polymers generally do not chemically interact with the bioactive compound. Most of the polymers used for encapsulation are biocompatible and biodegradable, ensuring safe integration with biological systems. Furthermore, the application of these materials as encapsulating agents enhances important properties such as the solubilization and/or dispersion of hydrophobic drugs or bioactive compounds like CBD in aqueous media [23].

Despite these advantages, certain challenges remain. Some polymers may exhibit instability under variable pH or enzymatic conditions, and scaling up encapsulation processes for industrial production can be complex. Moreover, while encapsulation of cannabinoids has been demonstrated, systematic studies focusing on the interaction of CBD with diverse polymeric matrices are still scarce. Looking ahead, future perspectives involve the development of stimuli-responsive polymers capable of releasing CBD in response to changes in pH, temperature, or enzymatic activity, thereby improving precision delivery. Additionally, hybrid nanocarriers, combining polymers with lipids or inorganic materials, are emerging as innovative platforms to further enhance the stability, release control, and therapeutic efficacy of cannabinoid-based formulations.

## 3. Polymeric Matrix Systems: Fundamentals and Classification

A wide range of polymeric materials with functional properties is employed across industrial and research fields. In pharmaceutical and cosmetic applications, the preference is often given to biocompatible and biodegradable polymers due to their safety and environmental advantages. The selection of a specific polymer depends primarily on the intended application and the physicochemical requirements of the target formulation. Although polymers can be classified in numerous ways, the most widely accepted approach categorizes them according to their origin as either natural or synthetic. Both classes have been extensively explored in the development of drug and bioactive compound delivery systems.

### 3.1. Natural vs. Synthetic Polymers

In drug delivery research, both natural and synthetic polymers have been investigated, with one of the primary objectives being to enhance compound stability both during storage and after administration into biological systems. Natural polymers are produced biologically by plants or other organisms, whereas synthetic polymers are manufactured or chemically modified in controlled laboratory conditions [24]. Natural polymers commonly applied in the encapsulation of pharmacological or bioactive compounds include chitosan, cellulose, alginates, gums, and pectins [25,26,27]. In recent years, synthetic polymers have gained increasing attention as efficient alternatives for encapsulation. Examples reported in the literature include polylactic acid (PLA), polymethyl methacrylate (PMMA), vinyl acetate (VAc), and polyvinyl alcohol (PVA), among others [25,28].

Both natural and synthetic polymers offer significant advantages in encapsulation processes, such as protection of the bioactive compound against environmental conditions (temperature, humidity, light, oxygen), enhancement of release kinetics within the biological system [29], and, more recently, the potential to engineer polymeric matrices at the nanometric scale to improve delivery performance [30].

It is worth noting that within both natural and synthetic categories, polymers with intrinsic biocompatible and biodegradable properties are available features that are highly desirable in the design of delivery systems for bioactive compounds [31]. There are distinct differences between natural and synthetic polymers that present both advantages and disadvantages depending on the application. On one hand, natural polymers have been cited as efficient alternatives for encapsulation; however, their high biodegradability, structural complexity, and sometimes limited stability can be seen as either advantages or drawbacks, depending on the intended use. Nonetheless, they generally maintain a high standard of bioavailability and biocompatibility [24].

Conversely, synthetic polymers have raised concerns due to their origin and reports of adverse reactions [32]. Many of them exhibit low biocompatibility in biomedical contexts; however, recent advances have enabled the structural modification of these materials to mimic the biocompatibility and biodegradability of their natural counterparts. Studies have demonstrated that synthetic polymeric matrices can achieve high encapsulation efficiency and controlled release, making them competitive options for advanced delivery platforms [33]. Overall, the choice between natural and synthetic polymers is not absolute but rather dictated by the desired balance between stability, biodegradability, biocompatibility, and manufacturing feasibility. This consideration becomes particularly relevant in the context of encapsulation methods, where the selection of the polymeric carrier is critical to achieving optimal performance in terms of protection, release profile, and targeted delivery.

### 3.2. Biodegradable and Biocompatible Matrices

In the pharmaceutical and biomedical fields, the use of biocompatible and biodegradable polymeric materials has been developed, as these are critical factors in the production of functional products and medical devices [34].

Some of the main properties of polymeric materials include high strength and durability; however, the increased consumption and production of plastic products have led to an alarming rise in waste generation worldwide. As a result, in recent decades, there has been a shift toward the use of biodegradable materials capable of breaking down under environmental conditions [35] in material production. A notable drawback of certain biodegradable materials is the absence of accelerated degradation mechanisms, making the modification of materials an emerging alternative. In encapsulation research involving polymeric materials, biodegradable polymers are often employed even when the application is not strictly biomedical. This approach is primarily aimed at promoting more sustainable production practices and minimizing waste generation.

As previously mentioned, plastics used in compound encapsulation are strictly biocompatible. This term refers to materials capable of supporting cellular behaviors, molecular signaling, and even regeneration without causing adverse effects on the host [36]. Currently, organizations such as the FDA (Food and Drug Administration) and EMA (European Medicines Agency) certify the use of certain polymeric materials for applications in food, pharmaceutical, cosmetic, and even technological sectors.

## 4. Encapsulation Techniques and Technologies

Encapsulation technologies and techniques for pharmaceutical compounds and natural components have been extensively studied in recent years. Multiple alternatives exist for encapsulating compounds using both synthetic and natural polymers.

### 4.1. Encapsulation by Emulsion Solvent Evaporation

A basic emulsion consists of an oil phase and an aqueous phase, stabilized by low molecular weight surfactants and high molecular weight polymers. This approach is mainly applied in the food, cosmetic, and pharmaceutical industries [37]. The solvent evaporation technique in emulsions, developed in the 1970s, is widely used to generate micro- and nanospheres with biocompatible polymeric materials. Unlike other encapsulation methods, such as spray drying, this technique does not require high energy consumption, only constant stirring and stable environmental conditions [38].

**Figure 3:**
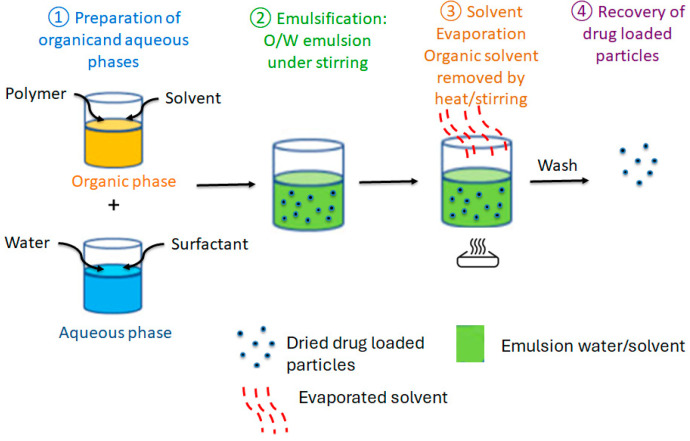
Emulsion solvent evaporation method for drug encapsulation.

The principle of this method (Figure 3) is based on conventional emulsions. However, solvents and sometimes cosolvents are used to improve the solubility of hydrophilic compounds in the oil phase, thereby increasing encapsulation efficiency [39]. This method can be applied both in conventional oil-in-water emulsions and in emulsions without an aqueous phase, which is particularly useful for encapsulating compounds that may degrade or dissolve in water [40]. Once the compounds are dissolved in the polymeric oil phase, they are dispersed in the aqueous phase to form an emulsion. Finally, solvents are removed by rotary evaporation or other techniques, leading to the spontaneous formation of polymeric matrices [37].

In recent years, the solvent evaporation method has been extensively applied for CBD encapsulation, showing its versatility in the pharmaceutical and biomedical fields. For example, David et al. [41] encapsulated CBD in PLGA microparticles via oil-in-water emulsification and solvent evaporation, reporting spherical particles with narrow size distribution and encapsulation efficiencies around 52%, later incorporated into hydrogels with sustained antimicrobial activity against S. aureus. Similarly, Villate et al. [42] evaluated extract-to-polymer ratios for cannabis extracts rich in CBD encapsulated in PLGA nanoparticles, highlighting the effect of formulation parameters on drug loading and release kinetics. These recent studies confirm that solvent evaporation remains a robust and adaptable method, capable of producing CBD-loaded carriers with tunable properties, high encapsulation efficiency, and promising therapeutic outcomes.

Freire et al. [8] developed Poly(butylene succinate) (PBS) nanoparticles loaded with CBD using a modified double emulsion/solvent evaporation method to overcome CBD’s low solubility and bioavailability. The nanoparticles (~175 nm) showed a biphasic release pattern with an initial burst followed by sustained release, indicating efficient encapsulation. Biological tests confirmed that CBD-PBS nanoparticles preserved the anticancer activity of free CBD while sparing normal fibroblasts. Incorporating CBD into the PBS matrix enhanced its chemical stability and dispersibility, preventing premature degradation and promoting controlled, bioavailable release. Also, Perez de la Ossa et al. [43] prepared Poly-ε-caprolactone (PCL) microspheres loaded with CBD using the oil-in-water emulsion–solvent evaporation method to overcome CBD’s high lipophilicity and chemical instability. The process yielded spherical microparticles (20–50 μm) with nearly 100% encapsulation efficiency, ensuring uniform drug distribution within the polymeric matrix. In vitro release tests showed a sustained release of CBD over 10 days, contrasting with the rapid degradation observed in free drug solutions. Cytotoxicity assays confirmed that encapsulated CBD retained and even enhanced its antitumoral effect, reducing the viability of MDA-MB-231 breast cancer cells by ≈60% after 7 days, compared with ≈50% for the free compound.

### 4.2. Encapsulation by Electrospinning Process

Electrospinning is another encapsulation technique for active compounds that has gained significant interest in recent years. The literature reports its application for the encapsulation of bioactive compounds, offering advantages such as operation at low temperatures, production of materials with large surface areas, high encapsulation efficiency, and cost-effectiveness. Additionally, it is versatile and easily scalable [44].

**Figure 4:**
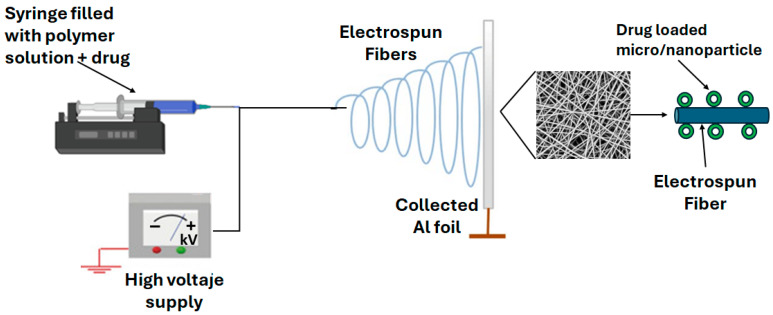
Electrospinning process for drug loading in polymeric fibers.

This technique is an electrohydrodynamic process (Figure 4) in which a polymer solution is ejected under an applied electric field, generating polymeric matrices capable of encapsulating compounds in micro- or nanoscale fibers or capsules [45]. One of the main advantages of electrospun matrices is their high porosity and functional surface-to-volume ratio, which is highly beneficial for encapsulation [46]. The methodology is relatively simple: a polymer solution (ethanol and other solvents have been reported) or a polymer emulsion containing the active compound is introduced into a syringe and deposited onto a high-voltage collector, typically metallic plates or rotating drum collectors [47]. Critical parameters affecting the final structure of the material include polymer solution concentration, solvent type, and applied voltage, as these factors directly influence morphology and encapsulation efficiency [48].

Recent studies have highlighted the potential of electrospinning for the encapsulation and delivery of cannabidiol (CBD), offering versatile platforms for oral and transdermal administration. Andriotis et al. [49] successfully developed water-soluble electrospun fibers for oral CBD delivery, employing hydrophilic polymer matrices that provided rapid dissolution and improved bioavailability compared to conventional formulations. Their work demonstrated that fiber morphology and polymer selection play critical roles in enhancing the solubility of lipophilic cannabinoids. More recently, Cruz et al. [50] advanced this concept by introducing a wearable, ultralow-power, and needleless electrospinning device capable of fabricating CBD-loaded patches directly on flexible substrates. This approach not only ensured efficient encapsulation but also opened new avenues for personalized, on-demand transdermal drug delivery systems.

### 4.3. Supercritical Fluid Processing

Supercritical fluids (SCFs), particularly carbon dioxide (CO_2_) with solvents, have been studied for the extraction and encapsulation of compounds. Depending on the cosolvent used, supercritical fluid technologies can generate FDA-approved products [51]. This method combines the solubilization capacity of liquids with the mass transport properties of gases [52]. Supercritical CO_2_ is widely employed as a solvent due to its gas-like diffusivity and liquid-like solubility, properties that allow efficient penetration into the target material for extraction or encapsulation. Encapsulation using a polymeric barrier through SCF technology helps preserve compounds, reduce oxidation, and maintain their structural and organoleptic properties [53]. Several SCF encapsulation methods exist, with the most recognized being: Rapid Expansion of Supercritical Solutions (RESS), Supercritical Solvent Impregnation (SSI), and Supercritical Fluid Extraction of Emulsions (SFEE) [54].

These methodologies are novel and could be promising options to encapsulate CBD, although, based on the available literature, no studies specifically applying RESS, SSI, or SFEE to CBD have been reported. In RESS, the solute dissolves in supercritical CO_2_ and, upon sudden expansion through a nozzle, the sharp density drop drives extreme supersaturation, burst nucleation, and precipitation of ultrafine, high-purity particles; a practical limitation is the low solubility of some solutes in CO_2_, which can be mitigated with cosolvents (e.g., ethanol). SSI technique leverages the solubility of the active in supercritical CO_2_ and polymer swelling, CO_2_ diffuses into the polymeric matrix carrying the active, and depressurization releases CO_2_, yielding a homogeneous distribution within the solid; it is gentle, energy-lean, and minimizes waste, making it attractive when loading films or shaped parts. SFEE starts from an emulsion in which the polymer and the active are in the organic phase; supercritical CO_2_ acts as an antisolvent/extractant to rapidly remove the organic solvent, inducing precipitation of micro-/nanocapsules with controlled size distributions, often achieving cleaner solvent removal and lower energy demand than spray drying or solvent evaporation. The most similar work to date was reported by Baldino et al. [23], who produced CBD nanoparticles embedded in PVP microparticles via supercritical CO_2_-assisted atomization (SAA), tuning the CBD/PVP ratio and total solids to control the size. They reported nanoparticles as small as 33 nm and a marked acceleration of dissolution (bulk CBD ≈ 240 min vs. ≈20 min for ~55 nm NPs), concluding that the nano-in-micro architecture substantially enhances dissolution rate and, by implication, potential bioavailability.

## 5. CBD–Polymer Interactions: Mechanistic Insights

Currently, numerous studies focus on the encapsulation of bioactive compounds. However, the interest in encapsulating phytocannabinoids, particularly CBD, has grown due to its therapeutic potential in treating diseases such as cancer, anxiety, and depression. At the same time, CBD has been widely employed to explore new encapsulation strategies, aiming at the development of efficient polymeric materials that provide protection against degradation and allow controlled release.

### 5.1. Chemical Bonding vs. Physical Entrapment

CBD–polymer interactions can mainly be classified into two mechanisms: chemical bonding and physical entrapment. In the case of chemical bonding, CBD can be covalently linked to the polymer or interact through hydrogen bonds or ionic forces, providing higher stability against presystemic release. Although several drug delivery systems based on non-covalent bonds have been designed, covalent polymer-drug conjugation is often considered more advantageous for encapsulation [55]. Both natural and synthetic polymers have been employed to establish such interactions, but the design of polymeric carriers requires considering several factors. The material should be biocompatible, biodegradable, and non-immunogenic, while also possessing functional groups that enable interaction with the compound. For polymers that do not undergo complete degradation in vivo, the molecular weight should remain below 50,000 g/mol (renal threshold) [56]. Various polymeric materials functionalized with drugs have already been approved for clinical applications. Among them, poly(ethylene glycol) (PEG) has been identified as a promising candidate, as its derivatives can be conjugated with active compounds to improve solubility and modulate plasma release profiles [57].

On the other hand, physical entrapment is the most common encapsulation mechanism. In this case, the bioactive compound is dispersed in the polymer matrix or enclosed within structures such as micelles, liposomes, or polymeric nanoparticles. While this strategy is simpler and avoids chemical modification of the active compound, it may present limitations in terms of long-term stability, since the compound can migrate or be prematurely released under adverse environmental conditions. This mechanism has been extensively studied for CBD encapsulation using synthetic polymeric matrices, oleosomes, proteins, and other carriers [8,58,59].

Recent advances in CBD encapsulation illustrate how both chemical bonding and physical entrapment strategies can be tailored depending on the intended application. For example, Andriotis et al. [8] demonstrated that electrospun water-soluble polymeric fibers enable effective incorporation of cannabinoids, enhancing their dissolution rate and stability through a matrix that maintains CBD in an amorphous form. This study exemplifies how physical entrapment within polymeric nanofibers avoids direct chemical modification while still achieving improved bioavailability. Similarly, Ma et al. [58] reported on protein-based nano-delivery systems that rely on hydrophobic interactions and hydrogen bonding to trap CBD molecules, showing promising results in terms of controlled release and gastrointestinal protection. In contrast, systems that integrate covalent conjugation can provide additional stability against premature degradation or release. Freire et al. [8] highlighted this by comparing synthetic polymer carriers where CBD was either physically dispersed or chemically bound, concluding that chemical conjugation extended retention time and reduced burst release, though at the expense of more complex processing steps. Taken together, these studies underscore that while physical entrapment approaches dominate due to their simplicity and versatility, there is increasing recognition of the benefits of combining or tuning both mechanisms. By selecting polymers with tailored functionalities, whether to form hydrogen bonds, facilitate covalent attachment, or enable nanoscale entrapment, researchers can design encapsulation platforms that optimize CBD’s solubility, stability, and pharmacokinetic performance.

### 5.2. Controlled Release Behavior and Kinetics

The release of CBD from polymeric matrices directly depends on the transport and degradation mechanisms of the system. The main processes described include diffusion of the compound through the matrix, polymer erosion, and combined mechanisms. Several studies apply mathematical models to describe release kinetics, the most common being the zero-order and first-order models, along with the Higuchi (diffusion-controlled) and Korsmeyer–Peppas (mixed diffusion and erosion) models. More recent approaches, including the Weibull, Peppas–Sahlin, and Hopfenberg models, have been proposed to more accurately describe complex non-Fickian or anomalous release behaviors observed in modern nanostructured carriers. These models incorporate additional parameters accounting for polymer relaxation, matrix geometry, and heterogeneous diffusion pathways, providing a better fit to experimental release data for multi-phase systems [60,61,62]. For CBD, release kinetics have been evaluated using matrices such as poly(butylene succinate) (PBS), where both Higuchi and Korsmeyer–Peppas models accurately describe diffusion-driven release [8]. The design of systems capable of modulating release makes it possible to maintain controlled plasma concentrations of CBD over time and extend its therapeutic efficacy in neurological, inflammatory, or anxiolytic applications.

Recent studies of CBD-controlled release underline the convergence of diffusion- and degradation-mediated mechanisms with new carrier designs. Lozza et al. [63] developed in situ forming PLGA implants, demonstrating reliable CBD release for a month, aligning well with sustained therapeutic needs. Complementing this, Morakul et al. [64] encapsulated cannabidiol within nanostructured lipid carriers (NLCs), which greatly enhanced CBD’s chemical stability and offered controlled lipid-mediated release profiles. Meanwhile, Toncheva-Moncheva and colleagues [65] introduced cinnamyl-modified polyglycidol/poly(ε-caprolactone) block copolymer micelles, achieving significantly higher encapsulation efficiency and notably prolonged release compared to non-functionalized counterparts. Additionally, David et al. [41] tackled CBD microparticle delivery by embedding CBD-loaded PLGA microparticles into porous scaffolds, thereby modulating the release through combined scaffold diffusion and polymer erosion mechanisms. Collectively, these studies illustrate diverse strategies, ranging from implantable PLGA matrices and lipidic carriers to functionalized block copolymers and scaffold-stabilized microparticles, that enhance control over CBD release. By leveraging materials that tailor diffusion, degradation, and carrier architecture, these platforms exemplify how CBD release kinetics can be optimized to maintain therapeutic levels over extended periods for neurological, inflammatory, or anxiolytic treatments.

### 5.3. Influence of Polymer Characteristics on Drug Loading and Release

The intrinsic properties of the polymer play an important role in encapsulation efficiency and release behavior. Therefore, compatibility between the matrix and the active compound must be considered. For example, polymers such as PLGA, which are hydrophobic [66], exhibit high affinity for hydrophobic compounds, whereas their conjugation with hydrophilic systems may reduce loading capacity but promote faster release. Another essential factor is polymer biodegradation. Although a high degradation rate may be desirable from an environmental perspective, in drug delivery, it must be carefully controlled to balance mechanical stability and release rate. Excessively biodegradable polymers can negatively affect both drug release and the mechanical properties of the matrices [67].

Additional factors relevant to drug release from polymeric systems include toxicity, carcinogenicity, and pharmacokinetics [68]. While these properties are more closely associated with the physicochemical nature of the material, they can be significantly altered when the polymer is functionalized with an active compound. As previously mentioned, structural modifications introduced by polymer and drug conjugation may strongly influence release behavior [56].

## 6. Material Performance and Characterization Approaches

The synthesis of the polymeric matrix is the main step in compound encapsulation; however, its physical, chemical, and structural evaluation is the most important part of developing encapsulating systems, as it is necessary to identify the properties that may provide advantages or disadvantages during their application. Characterization techniques have been the most effective tools to evaluate the physicochemical and structural properties of polymeric, ceramic, and even metallic systems. Their application is not only useful for material evaluation but also for optimization and modification during production [69]. For example, X-ray diffraction (XRD) provides information on crystalline structure, grain size, and composition; Fourier-transform infrared spectroscopy (FTIR) identifies chemical groups, molecular structure, and surface composition; thermogravimetric analysis (TGA) determines the mass composition of composite materials; and scanning electron microscopy (SEM), one of the most advanced techniques, allows the evaluation of morphology, microstructure, texture, and dispersion [70].

### 6.1. Morphology and Encapsulation Efficiency

Several studies on CBD encapsulation with organic and inorganic systems have been published. Depending on the coating matrix and application, certain characterization methods may be more suitable than others. Microscopy techniques such as SEM and TEM enable the evaluation of morphology and composition at high resolution, allowing the identification of particle dimensions or surface microstructure. In encapsulation, this is particularly relevant for assessing modifications in the matrices and understanding the physical pathways that occur during compound loading.

Dernaika et al. [71] encapsulated CBD in zeolite particles for oral administration by impregnation. SEM images revealed the morphology of zeolite particles before and after CBD loading, showing no significant structural changes, which suggested that loading occurred within the pores rather than on the nanoparticle surface. Freire et al. [8] synthesized polybutylene succinate (PBS) nanoparticles loaded with CBD using a modified double emulsion/solvent evaporation technique. TEM analysis confirmed the results observed by DLS and zeta potential measurements, showing polydispersity and particle dispersion with PBS nanoparticles equal to or smaller than 178 nm.

Baldino et al. [23] coated CBD nanoparticles with polyvinylpyrrolidone (PVP) using the supercritical fluid (CO_2_) encapsulation technique by assisted atomization. SEM analysis revealed regular spherical structures in CBD-PVP microparticles at different concentrations, with particle sizes equal to or smaller than one micron. The authors reported that compound–polymer concentrations affected particle size but not morphology.

### 6.2. Thermal Properties

The evaluation of thermal properties in polymeric systems used for CBD or other compound encapsulation is essential to determine their stability, performance, and potential application in therapeutic formulations. Thermal characterization techniques, particularly Differential Scanning Calorimetry (DSC) and Thermogravimetric Analysis (TGA), identify transitions such as glass transition, melting, and decomposition temperatures, which are directly related to encapsulation efficiency and compound stability under different environmental conditions.

As mentioned, DSC is useful for identifying the behavior of polymeric materials with increasing temperature. This analysis is important to determine polymer transition temperatures. In compound encapsulation with polymeric materials, this technique has been used not only to recognize the polymer’s glass transition temperature (Tg) but also to evaluate the polymer’s functionality as an encapsulating matrix. Ansari and Alshahrani [72] encapsulated Baricitinib, an antirheumatic drug with low aqueous solubility and poor bioavailability for oral administration, with poly(lactic-co-glycolic acid) (PLGA) copolymer. Using DSC, they analyzed PLGA nanoparticles with encapsulated drug and pure drug. They identified a higher Tg than that reported in the literature for the copolymer, as well as the absence of the melting peak of the matrix with the drug. They concluded that the drug was encapsulated and exhibited an amorphous molecular dispersion within the copolymer.

On the other hand, material characterization by TGA evaluates composition through weight loss with increasing temperature. This technique is useful for evaluating various parameters such as material composition and degradation temperatures of compounds. For encapsulation, TGA has proven valuable, as its application has been reported in the literature to calculate encapsulation percentage and to evaluate thermal stability [73]. Sreekanth [74] developed the encapsulation of curcumin in crosslinked sodium alginate (SA) and montmorillonite (MMT) microspheres to improve its release. TGA identified the behavior of MMT, curcumin, and both loaded and unloaded spheres. Thermograms allowed the identification of compound weight percentages in the microspheres, degradation temperatures of each component, and a significant increase in the thermal stability of encapsulated curcumin within the microbeads.

### 6.3. Swelling Behavior, Degradation, and Release Profile

The thermal and structural properties of polymeric systems not only determine the stability of encapsulated compounds under environmental conditions but are also directly linked to key factors such as degradation, release profile, and swelling. The hydrophilic or hydrophobic nature of polymers influences the degree of swelling depending on the medium. Several hydrophilic polymer formulations for prolonged drug release have been reported. One advantage of this type of system is its ability to regulate drug release and diffusion rates in aqueous environments [75].

It is important to distinguish that the swelling and release mechanisms differ markedly between hydrogels and solid or nanoparticulate polymer matrices. Hydrogels possess crosslinked, three-dimensional networks that absorb significant amounts of water, where swelling is governed by osmotic pressure and elastic retraction forces within the polymer network. This volumetric expansion allows drug diffusion through hydrated channels and often follows Fickian or anomalous transport. In contrast, compact polymeric matrices or nanoparticles typically do not exhibit macroscopic swelling; their release behavior is mainly determined by molecular diffusion of the drug through the polymer phase, polymer relaxation, or gradual degradation and erosion of the carrier. Therefore, while both systems can achieve controlled release, the underlying physicochemical mechanisms are fundamentally different.

Swelling behavior is often studied in hydrogels, a class of polymers widely applied in the biomedical field. Structurally, hydrogels are formed by interconnected three-dimensional networks that allow fluid absorption and retention [76]. They have been used in tissue engineering as matrices for regeneration, since their hydrophilic structure supports cellular, metabolic, and fluid diffusion [77]. Their application in drug delivery has also been widely reported [78,79]. In smaller polymeric matrices such as compacted micro- or nanoparticles, compound release is more complex. When the polymer is put in contact with the medium, a gel layer forms on the surface; this is the swelling behavior, through which polymer chains allow controlled drug diffusion [80].

Swelling behavior is directly related to release: depending on whether the matrices are hydrophilic or hydrophobic, the polymer undergoes dissolution or degradation. Upon penetration by the aqueous medium, swelling reduces the glass transition temperature (Tg), forming a gel-like consistency, while osmotic pressure enhances transport and the formation of an entangled polymer solution, facilitating compound release. This release process can be modeled according to Fick’s second law of diffusion [81]. After polymer hydration or swelling and drug release, polymer dissolution or degradation occurs. However, depending on the polymeric matrix, drug release may instead occur through channels within the structure, allowing compound diffusion without complete matrix degradation [80].

Hydrogels play a crucial role in enhancing both the solubility and the stability of cannabidiol. Cui et al. [82] prepared modified PVA hydrogels and encapsulated CBD to study its aqueous release. They observed good mechanical strength and stiffness of hydrogels for easy manipulation. The CBD in the hydrogels was continuously released into an aqueous environment and showed radical scavenger activity for at least 24 h, improving CBD stability. The three-dimensional, hydrophilic polymeric network allows CBD molecules to be dispersed at the nanoscale within hydrated polymer domains, effectively increasing their apparent solubility and bioavailability. This molecular confinement reduces aggregation and facilitates uniform distribution throughout the matrix. Moreover, the hydrogel structure acts as a protective microenvironment for CBD, decreasing interaction with external factors such as light, oxygen, and temperature changes. The limited diffusion of oxygen and reactive species through the gel, together with hydrogen bonding and polymer–CBD interactions (e.g., hydroxyl and carboxyl groups), retards oxidation and isomerization reactions responsible for CBD instability. Several studies have demonstrated that encapsulating cannabinoids within polymeric or lipid hydrogels significantly prolongs shelf life, reducing degradation compared with free CBD solutions. Therefore, the hydrogel matrix not only improves aqueous compatibility but also provides chemical stabilization through physical encapsulation and controlled molecular mobility.

Zhang et al. [83] designed a multifunctional hydrogel for a contact lens for corneal alkali burn healing. Hydrogel consisted of citric acid/CBD/quaternary ammonium chitosan (QCS)/polyvinyl alcohol. This structure enabled a pH-responsive release of therapeutic agents: under alkaline conditions, the hydrogel released ≈52% of citric acid and 59% CBD within 24 h. The results showed that the inclusion of CBD in the hydrogel not only provided anti-inflammatory and antioxidant effects but also improved the chemical stability of CBD under alkaline pH, as the polymeric matrix protected CBD molecules from rapid oxidation and hydroxide ion attack. Consequently, the hydrogel efficiently neutralized local alkalinity, scavenged hydroxyl radicals, reduced inflammatory cytokine expression, and inhibited bacterial growth.

Table 1 shows different materials studied for CDB encapsulation, along with applications and the most important results found. The table shows recent advances in CBD encapsulation using diverse polymeric systems and techniques, highlighting their applications and performance outcomes. It shows that encapsulation methods such as double and oil-in-water emulsions, solvent evaporation, Pickering emulsions, and supercritical CO_2_ atomization have been successfully employed for controlled drug release, topical delivery, and protection of bioactive compounds. Biopolymers like poly(butylene succinate), polycaprolactone (PCL), polyvinylpyrrolidone (PVP), chitosan, and whey protein–based systems demonstrated high efficiency in stabilizing CBD, often achieving controlled or sustained release over several hours to days. Notably, formulations using chitosan and gum arabic achieved over 95% CBD recovery and optimal skin absorption, while PVP-based nanoparticles enabled rapid dissolution within 20 min. In contrast, PLGA-based in situ implants and PCL microspheres provided prolonged release suitable for anticancer therapy. Overall, the comparison reveals that emulsion-based and biodegradable polymer systems effectively improve CBD bioavailability, stability, and therapeutic performance, tailoring the release kinetics to specific oral, topical, or implantable delivery routes.

## 7. Challenges, Gaps, and Future Perspectives

Despite the promising outcomes of polymeric encapsulation, several challenges remain. Stability and scalability are the main concerns: CBD is prone to rapid degradation, and although polymeric matrices improve resistance, ensuring long-term stability under industrial processing and storage conditions requires further research. Scale-up of nanostructured systems is also hindered by high production costs, batch variability, and limited reproducibility of encapsulation efficiency. Regulatory and standardization hurdles also present critical barriers. Differences in global regulations concerning CBD products complicate quality assurance, safety evaluation, and market approval. Standardized protocols for assessing encapsulation efficiency, release kinetics, and biocompatibility are urgently needed to bridge laboratory advances with clinical translation.

Beyond scientific and technological challenges, the legal status of CBD remains a decisive barrier for the clinical and industrial translation of encapsulation systems. Regulations governing cannabidiol vary widely across countries. In the United States and the European Union, only Epidiolex^®^ is fully approved as a pharmaceutical product by the FDA and EMA, respectively. Most other CBD formulations are classified as dietary supplements or cosmetics, lacking formal authorization for medical claims. These regulatory inconsistencies complicate large-scale manufacturing, clinical evaluation, and market entry of novel polymeric or nanostructured carriers. Consequently, harmonization of international legislation, clearer definitions of product categories, and robust pharmacovigilance frameworks are essential to accelerate the safe and standardized deployment of CBD-based therapeutic delivery systems.

Looking ahead, emerging materials and smart polymers represent a major frontier. Stimuli-responsive polymers capable of modulating CBD release in response to pH, temperature, or enzymatic triggers could enable precision therapies. Hybrid systems integrating polymers with lipids, proteins, or inorganic carriers may further enhance targeted delivery and reduce systemic side effects. Also, advances in computational modeling and machine learning may accelerate the design of optimized encapsulating matrices by predicting stability, drug–polymer interactions, and release profiles. Overall, bridging the current knowledge gaps requires interdisciplinary collaboration between material scientists, pharmacologists, and regulatory bodies to enable the safe, scalable, and effective integration of polymer-CBD systems into mainstream therapeutics.

## 8. Conclusions

Polymeric encapsulation is a promising technique for enhancing the therapeutic potential of cannabidiol and its derivatives by addressing key challenges of solubility, stability, and bioavailability. Both natural and synthetic polymers provide adaptable carriers, ensuring protection against environmental degradation and enabling controlled release through mechanisms such as diffusion and polymer erosion. Techniques like emulsion solvent evaporation, electrospinning, and supercritical fluid processing have proven effective in generating nanoscale delivery systems with improved pharmacological performance. Characterization tools including SEM, TEM, DSC, and TGA remain indispensable for optimizing system design, validating encapsulation efficiency, and ensuring reproducibility. Nevertheless, critical barriers persist. Industrial scalability, regulatory standardization, and long-term stability remain unresolved, limiting the transition of laboratory successes into clinical practice. To move forward, it is essential to establish harmonized testing protocols, reduce production variability, and develop cost-effective methods for large-scale manufacturing. In parallel, the exploration of stimuli-responsive and multifunctional polymers offers a pathway to more precise, patient-specific therapies.

In conclusion, polymeric encapsulation represents a transformative approach to harnessing CBD’s therapeutic potential, bridging its molecular limitations with advanced material science solutions. By integrating novel polymer design, predictive modeling, and standardized evaluation frameworks, future research can accelerate the translation of CBD-polymer systems from experimental platforms to real applications, enhancing patient outcomes and expanding therapeutic options.

## Figures and Tables

**Table 1:** Summary of Encapsulation Techniques, Polymeric Systems, Applications, and Main Results Achieved for Cannabidiol (CBD) Delivery.

Encapsulation Technique	Polymeric System Used	Applications	Main Results Achieved
Double emulsion/solvent evaporation technique [8]	Poly (butylene succinate) (PBS)	Anticancer application	Controlled release of CBD in the first 3–5 h at approximately 50% and slow release after the first hours 75% in 72 h.
Drop-by-drop anti-solvent precipitation method [84]	Zeina and Zein-whey protein (WP)	Protection of compounds in food processing	Controlled release of CBD in vitro in simulated gastric and intestinal fluid, with release around 75% in CBD/Zein and 92% in CBD/Zein-WP.
Oil-water emulsion [59]	Whey protein (WP) and WP-maltodextrin	Emulsion-based delivery systems	Using WP-MD as a stabilizer with a 50:50 medium-chain triglyceride (MCT) and long-chain triglyceride (LCT) ratio produced more stable emulsions, suitable for long-term CBD preservation in 16 days at 55 °C.
Pickering emulsión (PEs) [85]	High and low DDA chitosan/gum Arabic	Topical delivery of CBD	CH/GA particles containing high-DDA chitosan showed good affinity and adherence to skin cells. Both formulations achieved over 95% CBD recovery, regarded as optimal under OECD ex vivo skin absorption guidelines. The skin absorbed roughly 2.9% and 4.3% of the total CBD for high- and low-DDA chitosan systems, respectively.
Supercritical CO_2_ atomization [23]	Polivinylpyrrolidone (PVP)	Oral controlled-release system	CBD release powder fully dissolved in around 240 min, whereas the 55 nm CBD nanoparticle with PVP was completely released in just 20 min
Solvent exchange process [86]	Polycaprolactone (PCL)	Evaluation of anticancer activity	One of the formulations suppressed the proliferation and migration of MDA-MB-231 and 4T1 cells and demonstrated an antiangiogenic effect in in ovo models.
Oil-water emulsion [87]	Whey protein (WP) Whey protein-maltodextrin (WP-MD) WP-MD-Rosmarinic acid (RA)	Design emulsion systems that protect active substances from environmental conditions.	Results showed that WP-MD-RA was an efficient emulsifier, producing fine droplets and enhancing pH and salt stability. It provided the greatest CBD protection against UV and heat degradation and maintained a small particle size during storage at 4 °C.
Freeze–thaw method [82]	Conjugated systems of Poly(vinyl alcohol) (PVA), propylene glycol (PG) and vegetable glycerine (VG)	Controlled release into the system	CBD release was evaluated after 24 h. The PVA system released 60% of the CBD, while the PVA-PG and PVA-PG-VG systems released 65%, and the PVA-PG system released 75% of the encapsulated CBD.
Injectable Solid-in-Oil or In Situ Forming Implants [63]	PLA-202/203 and PLGA 502H/503	Production of in situ forming implants (ISFIs) for cancer application	PLGA 502 implants prepared with DMSO as the solvent and a CBD/polymer ratio of 5:100 (*w*/*w*) exhibited an initial release below 25% and nearly constant release over one month, a crucial period for cancer therapy applications.

## Data Availability

No new data was created.

## References

[1] Faiz M.B., Naeem F., Irfan M., Aslam M.A., Estevinho L.M., Ateşşahin D.A., Alshahrani A.M., Calina D., Khan K., Sharifi-Rad J. (2024). Exploring the therapeutic potential of cannabinoids in cancer by modulating signaling pathways and addressing clinical challenges. Discov. Oncol..

[2] Shah S.A., Gupta A.S., Kumar P. (2021). Emerging role of cannabinoids and synthetic cannabinoid receptor 1/cannabinoid receptor 2 receptor agonists in cancer treatment and chemotherapy-associated cancer management. J. Cancer Res. Ther..

[3] Challa S.K.R., Misra N.N., Martynenko A. (2021). Drying of cannabis—State of the practices and future needs. Dry. Technol..

[4] Kuzumi A., Yoshizaki-Ogawa A., Fukasawa T., Sato S., Yoshizaki A. (2024). The Potential Role of Cannabidiol in Cosmetic Dermatology: A Literature Review. Am. J. Clin. Dermatol..

[5] Selvaraj S., Nawfer N., Dharmawansa K.S., Redha A.A., Rupasinghe H.V. (2025). Recent advances in cannabidiol (CBD) extraction: A review of potential eco-friendly solvents and advanced technologies. Green Anal. Chem..

[6] Pillai S.K., Kera N.H., Kleyi P., de Beer M., Magwaza M., Ray S.S. (2024). Stability, biofunctional, and antimicrobial characteristics of cannabidiol isolate for the design of topical formulations. Soft Matter.

[7] Samara E., Bialer M., Mechoulam R. (1988). Pharmacokinetics of cannabidiol in dogs. Drug Metab. Dispos..

[8] Freire N.F., Cordani M., Aparicio-Blanco J., Sanchez A.I.F., Dutra L., Pinto M.C., Zarrabi A., Pinto J.C., Velasco G., Fialho R. (2024). Preparation and characterization of PBS (Polybutylene Succinate) nanoparticles containing cannabidiol (CBD) for anticancer application. J. Drug Deliv. Sci. Technol..

[9] Ortiz-Romero N., Ochoa-Martínez L.A., González-Herrera S.M., Rutiaga-Quiñones O.M., Gallegos-Infante J.A. (2021). Avances en las investigaciones sobre la encapsulación mediante gelación iónica: Una revisión sistemática. TecnoLógicas.

[10] Barbosa-Nuñez J.A., Espinosa-Andrews H., Cardona A.A.V., Haro-González J.N. (2025). Polymer-based encapsulation in food products: A comprehensive review of applications and advancements. J. Futur. Foods.

[11] Jose M.S., Sumathi S. (2025). A review of electrospun polymeric fibers as potential drug delivery systems for tunable release kinetics. J. Sci. Adv. Mater. Devices.

[12] George A., Shah P.A., Shrivastav P.S. (2019). Natural biodegradable polymers based nano-formulations for drug delivery: A review. Int. J. Pharm..

[13] Eagleston L.R.M., Kalani Yazd N.K., Patel R.R., Flaten H.K., Dunnick C.A., Dellavalle R.P. (2018). Cannabinoids in dermatology: A scoping review. Dermatol. Online J..

[14] Brighenti V., Protti M., Anceschi L., Zanardi C., Mercolini L., Pellati F. (2021). Emerging challenges in the extraction, analysis and bioanalysis of cannabidiol and related compounds. J. Pharm. Biomed. Anal..

[15] Tihăuan B.–.M., Onisei T., Slootweg W., Gună D., Iliescu C., Chifiriuc M.C. (2025). Cannabidiol—A friend or a foe?. Eur. J. Pharm. Sci..

[16] Fraguas-Sánchez A., Fernández-Carballido A., Martin-Sabroso C., Torres-Suárez A. (2020). Stability characteristics of cannabidiol for the design of pharmacological, biochemical and pharmaceutical studies. J. Chromatogr. B.

[17] Koryťáková A., Chatziadi A., Rohlíček J., Zmeškalová E., Beránek J., Šoóš M. (2025). Stability study and structural insights into cannabidiol cocrystals. CrystEngComm.

[18] Kosović E., Sýkora D., Kuchař M. (2021). Stability Study of Cannabidiol in the Form of Solid Powder and Sunflower Oil Solution. Pharmaceutics.

[19] Bini A., Salerno S., Protti S., Pollastro F., Profumo A., Morini L., Merli D. (2024). Photodegradation of cannabidiol (CBD) and Δ^9^-THC in cannabis plant material. Photochem. Photobiol. Sci..

[20] Jaidee W., Siridechakorn I., Nessopa S., Wisuitiprot V., Chaiwangrach N., Ingkaninan K., Waranuch N. (2022). Kinetics of CBD, Δ^9^-THC Degradation and Cannabinol Formation in Cannabis Resin at Various Temperature and pH Conditions. Cannabis Cannabinoid Res..

[21] Buerhop C., Stroyuk O., Mashkov O., Barabash A., Hauch J., Peters I. (2024). Polymer encapsulation impact on potential-induced degradation in PV modules revealed by a multi-modal field study. Sol. Energy Mater. Sol. Cells.

[22] Yi L., Shi L., Móczó J., Pukánszky B. (2024). Encapsulation of a drug into electrospun fibers spun from water soluble polymers to control solubility and release. Heliyon.

[23] Baldino L., Sarnelli S., Palazzo I., Scognamiglio M., Reverchon E. (2025). Production of cannabidiol nanoparticles loaded in polyvinylpyrrolidone microparticles by supercritical CO_2_ assisted atomization and dissolution enhancement effect. Adv. Powder Technol..

[24] Bhatia S. (2016). Natural Polymers vs. Synthetic Polymer. Natural Polymer Drug Delivery Systems.

[25] Rezazadeh A., Bazardeh M.E., Ghasempour Z., Kia E.M. (2025). Gelatin/pectin complex coacervation for encapsulation of microwave-assisted extraction of bioactive compounds from red onion skin. Int. J. Biol. Macromol..

[26] Tripty M.R., Nupur A.H., Jany J.F., Toma M.A., Mazumder A.R. (2025). Encapsulation of mango peel bioactive compounds in milk, gum acacia, and maltodextrin improves its stability. NFS J..

[27] Afra S., Koch M., Żur-Pińska J., Dolatshahi M., Bahrami A.R., Sayed J.E., Moradi A., Matin M.M., Włodarczyk-Biegun M.K. (2024). Chitosan/Nanohydroxyapatite/Hydroxyethyl-cellulose-based printable formulations for local alendronate drug delivery in osteoporosis treatment. Carbohydr. Polym. Technol. Appl..

[28] Secerli J., Adatepe Ş., Altuntas S., Topal G.R., Erdem O., Bacanlı M. (2023). In vitro toxicity of naringin and berberine alone, and encapsulated within PMMA nanoparticles. Toxicol. Vitr..

[29] Rezagholizade-Shirvan A., Soltani M., Shokri S., Radfar R., Arab M., Shamloo E. (2024). Bioactive compound encapsulation: Characteristics, applications in food systems, and implications for human health. Food Chem. X.

[30] Machtakova M., Thérien-Aubin H., Landfester K. (2021). Polymer nano-systems for the encapsulation and delivery of active biomacromolecular therapeutic agents. Chem. Soc. Rev..

[31] Rahul P.B., Tiwari R.K., Dash K.K., Sharma M. (2025). Recent advances in encapsulation of pomegranate peel extract and combination of wall materials: A review of encapsulation technologies, characterization and applications in the food industry. Sustain. Food Technol..

[32] Yuan M., Hu M., Dai F., Fan Y., Deng Z., Deng H., Cheng Y. (2021). Application of synthetic and natural polymers in surgical mesh for pelvic floor reconstruction. Mater. Des..

[33] Suhas P., Mahesh B., Divakara S., Nanjundaswamy G., Prasad C.M., Sionkowska A., Popat K.C., Gowda D.C. (2025). Synergistic approaches in natural and synthetic polymer blends for biomedical applications-A review. Eur. Polym. J..

[34] Zwicker P., Hornschuh M., Schmidt T., Schäfer J., Becker-Willinger C., Jochum M., Kramer A., Müller G. (2025). A biocompatible polylactide-ε-caprolactone polymer coated with poly(hexamethylene biguanide) displays antibacterial properties against slime-producing *S. epidermidis*. Mater. Adv..

[35] Moutinho L.G., Soares E., Oliveira M. (2025). Biodegradability assessment of cork polymer composites for sustainable packaging applications. Next Mater..

[36] Gu X., Ding F., Yang Y., Liu J. (2015). Tissue Engineering in Peripheral Nerve Regeneration. Neural Regeneration.

[37] Dai L., Zhan X., Wei Y., Sun C., Mao L., McClements D.J., Gao Y. (2018). Composite zein—Propylene glycol alginate particles prepared using solvent evaporation: Characterization and application as Pickering emulsion stabilizers. Food Hydrocoll..

[38] Kim B.K., Hwang S.J., Park J.B., Park H.J. (2002). Preparation and characterization of drug-loaded polymethacrylate microspheres by an emulsion solvent evaporation method. J. Microencapsul..

[39] Safari H., Felder M.L., Kaczorowski N., Eniola-Adefeso O. (2022). Effect of the Emulsion Solvent Evaporation Technique Cosolvent Choice on the Loading Efficiency and Release Profile of Anti-CD47 from PLGA Nanospheres. J. Pharm. Sci..

[40] Meng F., Wang S., Wang Y., Liu H., Huo X., Ma H., Ma Z., Xiong H. (2017). Microencapsulation of oxalic acid via oil-in-oil (O/O) emulsion solvent evaporation. Powder Technol..

[41] David C., de Souza J.F., Silva A.F., Grazioli G., Barboza A.S., Lund R.G., Fajardo A.R., Moraes R.R. (2024). Cannabidiol-loaded microparticles embedded in a porous hydrogel matrix for biomedical applications. J. Mater. Sci. Mater. Med..

[42] Villate A., Barreto G.P., Nicolás M.S., Aizpurua-Olaizola O., Olivares M., Usobiaga A. (2024). Development, Characterization and In Vitro Gastrointestinal Release of PLGA Nanoparticles Loaded with Full-Spectrum Cannabis Extracts. AAPS PharmSciTech.

[43] de la Ossa D.H.P., Ligresti A., Gil-Alegre M., Aberturas M., Molpeceres J., Di Marzo V., Suárez A.T. (2012). Poly-ε-caprolactone microspheres as a drug delivery system for cannabinoid administration: Development, characterization and in vitro evaluation of their antitumoral efficacy. J. Control. Release.

[44] Pires J.B., dos Santos F.N., Costa I.H.d.L., Kringel D.H., Zavareze E.d.R., Dias A.R.G. (2023). Essential oil encapsulation by electrospinning and electrospraying using food proteins: A review. Food Res. Int..

[45] Pires J.B., Fonseca L.M., Siebeneichler T.J., Crizel R.L., dos Santos F.N., Hackbart H.C.d.S., Kringel D.H., Meinhart A.D., Zavareze E.d.R., Dias A.R.G. (2022). Curcumin encapsulation in capsules and fibers of potato starch by electrospraying and electrospinning: Thermal resistance and antioxidant activity. Food Res. Int..

[46] Coelho S.C., Estevinho B.N., Rocha F. (2021). Encapsulation in food industry with emerging electrohydrodynamic techniques: Electrospinning and electrospraying—A review. Food Chem..

[47] Reksamunandar R.P., Edikresnha D., Munir M.M., Damayanti S. (2017). Khairurrijal Encapsulation of β-carotene in poly(vinylpyrrolidone) (PVP) by Electrospinning Technique. Procedia Eng..

[48] Alfonso I., Calvo-Correas T., Eceiza A., Claver A., Torresi S., García J.A., Zalakain I. (2025). Recycling bovine ear tags for phase change material encapsulation via electrospinning. Sustain. Mater. Technol..

[49] Andriotis E.G., Chachlioutaki K., Monou P.K., Bouropoulos N., Tzetzis D., Barmpalexis P., Chang M.-W., Ahmad Z., Fatouros D.G. (2021). Development of Water-Soluble Electrospun Fibers for the Oral Delivery of Cannabinoids. AAPS PharmSciTech.

[50] Cruz O.B., Lou L., Mohammed S.M.A.K., Murickan R.T., Benedetti L., Lin Y.-M., Dolmetsch T., Agarwal A. (2025). Wearable, Ultralow Power, and Needleless Electrospinning Equipment for Cannabidiol-Loaded Patch Fabrication. ACS Appl. Mater. Interfaces.

[51] Wenzel J., Samaniego C.S., Wang L., Burrows L., Tucker E., Dwarshuis N., Ammerman M., Zand A. (2017). Antioxidant potential of *Juglans nigra*, black walnut, husks extracted using supercritical carbon dioxide with an ethanol modifier. Food Sci. Nutr..

[52] Hoseini S.A., Vazifedoost M., Hajirostamloo B., Didar Z., Nematshahi M.M. (2025). Supercritical fluid extraction and encapsulation of Rivas (*Rheum ribes*) flower: Principal component analysis (PCA). Heliyon.

[53] Naziruddin M., Jawaid M., Elais R., Sanny M., Fouad H., Yusof N., Abdul-Mutalib N. (2023). Supercritical fluid extraction of torch ginger: Encapsulation, metabolite profiling, and antioxidant activity. J. King Saud Univ. Sci..

[54] Cerro D., Rojas A., Torres A., Villegas C., Galotto M.J., Guarda A., Romero J. (2023). Nanoencapsulation of food-grade bioactive compounds using a supercritical fluid extraction of emulsions process: Effect of operational variables on the properties of nanocapsules and new perspectives. LWT.

[55] Fuentes-Ríos D., Moya-Utrera F., Moreno J., Mesas C., Doña-Flores M., Sarabia F., López-Romero J.M., Melguizo C., Prados J. (2024). Synthesis, characterization and antitumor activity of a poly-4-Vinyl pyridine-co-cannabidiol polymer. Eur. Polym. J..

[56] Ulbrich K., Holá K., Šubr V., Bakandritsos A., Tuček J., Zbořil R. (2016). Targeted Drug Delivery with Polymers and Magnetic Nanoparticles: Covalent and Noncovalent Approaches, Release Control, and Clinical Studies. Chem. Rev..

[57] Rodrigues P.C., Beyer U., Schumacher P., Roth T., Fiebig H.H., Unger C., Messori L., Orioli P., Paper D.H., Mülhaupt R. (1999). Acid-sensitive polyethylene glycol conjugates of doxorubicin: Preparation, in vitro efficacy and intracellular distribution. Bioorganic Med. Chem..

[58] Ma Z., Bitter J.H., Boom R.M., Nikiforidis C.V. (2024). Encapsulation of cannabidiol in hemp seed oleosomes. Food Res. Int..

[59] Wang C., Zhang X., Zhao R., Freeman K., McHenry M.A., Wang C., Guo M. (2022). Impact of carrier oil on interfacial properties, CBD partition and stability of emulsions formulated by whey protein or whey protein-maltodextrin conjugate. LWT.

[60] Korzhikov-Vlakh V., Sinitsyna E., Stepanova M., Korzhikova-Vlakh E., Tennikova T. (2025). Comparison of Different Aliphatic Polyester-Based Microparticles as Protein Delivery Systems. Polymers.

[61] Lakshani N., Wijerathne H.S., Sandaruwan C., Kottegoda N., Karunarathne V. (2023). Release Kinetic Models and Release Mechanisms of Controlled-Release and Slow-Release Fertilizers. ACS Agric. Sci. Technol..

[62] Martín-Camacho U.d.J., Rodríguez-Barajas N., Sánchez-Burgos J.A., Pérez-Larios A. (2023). Weibull β value for the discernment of drug release mechanism of PLGA particles. Int. J. Pharm..

[63] Lozza I., Martín-Sabroso C., Torres-Suárez A.I., Fraguas-Sánchez A.I. (2024). In situ forming PLA and PLGA implants for the parenteral administration of Cannabidiol. Int. J. Pharm..

[64] Morakul B., Junyaprasert V.B., Sakchaisri K., Teeranachaideekul V. (2023). Cannabidiol-Loaded Nanostructured Lipid Carriers (NLCs) for Dermal Delivery: Enhancement of Photostability, Cell Viability, and Anti-Inflammatory Activity. Pharmaceutics.

[65] Toncheva-Moncheva N., Dimitrov E., Grancharov G., Momekova D., Petrov P., Rangelov S. (2023). Cinnamyl-Modified Polyglycidol/Poly(ε-Caprolactone) Block Copolymer Nanocarriers for Enhanced Encapsulation and Prolonged Release of Cannabidiol. Pharmaceutics.

[66] Sunoqrot S., Alkurdi M., Al Bawab A.Q., Hammad A.M., Tayyem R., Abu Obeed A., Abufara M. (2023). Encapsulation of morin in lipid core/PLGA shell nanoparticles significantly enhances its anti-inflammatory activity and oral bioavailability. Saudi Pharm. J..

[67] Wang X., Wang C., Chu C., Xue F., Li J., Bai J. (2024). Structure-function integrated biodegradable Mg/polymer composites: Design, manufacturing, properties, and biomedical applications. Bioact. Mater..

[68] Rojas K., Verdugo-Molinares M.G., Vallejo-Cardona A.A. (2024). Use of encapsulating polymers of active compounds in the pharmaceutical and food industry. Food Chem. Adv..

[69] Zhang S., Li L., Kumar A. (2008). Materials Characterization Techniques.

[70] Mourdikoudis S., Pallares R.M., Thanh N.T.K. (2018). Characterization techniques for nanoparticles: Comparison and complementarity upon studying nanoparticle properties. Nanoscale.

[71] Dernaika F., Halawy L., Zeaiter J., Kawrani S., Mroue D., Lteif A., Kourani S., Mehanna M., Abboud C., Mroueh M. (2024). Development and characterization of a zeolite based drug delivery system: Application to cannabidiol oral delivery. Heliyon.

[72] Ansari M.J., Alshahrani S.M. (2019). Nano-encapsulation and characterization of baricitinib using poly-lactic-glycolic acid co-polymer. Saudi Pharm. J..

[73] Saiyasombat W., Yimpetch C., Chathiran W., Chimasangkanan J., Srichamnong W. (2025). Effect of microencapsulation using β-cyclodextrin and β-glucan as coating agents on physicochemical properties and phytocannabinoids retention of cannabis flower oil extract. NFS J..

[74] Reddy O.S., Subha M., Jithendra T., Madhavi C., Rao K.C. (2021). Curcumin encapsulated dual cross linked sodium alginate/montmorillonite polymeric composite beads for controlled drug delivery. J. Pharm. Anal..

[75] Muhamad H., Ward A., Patel K., Williamson J., Blunt L., Conway B., Østergaard J., Asare-Addo K. (2024). Investigation into the swelling and dissolution behaviour of Polymer-Excipient blends of PEO Utilising dissolution imaging. Int. J. Pharm..

[76] Lohani A., Saxena R., Duarte J.G., Khan S., Figueiras A., Mascarenhas-Melo F. (2025). Tailored polymeric hydrogels for regenerative medicine and drug delivery: From material design to clinical applications. Int. J. Pharm..

[77] Carton F., Rizzi M., Canciani E., Sieve G., Di Francesco D., Casarella S., Di Nunno L., Boccafoschi F. (2024). Use of Hydrogels in Regenerative Medicine: Focus on Mechanical Properties. Int. J. Mol. Sci..

[78] Hu X., Liang R., Li J., Liu Z., Sun G. (2019). Mechanically strong hydrogels achieved by designing homogeneous network structure. Mater. Des..

[79] Ovando-Medina V.M., Reyes-Palacios G.A., García-Montejano L.A., Antonio-Carmona I.D., Martínez-Gutiérrez H. (2021). Electroactive polyacrylamide/chitosan/polypyrrole hydrogel for captopril release controlled by electricity. J. Vinyl Addit. Technol..

[80] Funk N.L., Fantaus S., Beck R.C.R. (2022). Immediate release 3D printed oral dosage forms: How different polymers have been explored to reach suitable drug release behaviour. Int. J. Pharm..

[81] Korelc K., Tzanova M.M., Larsson A., Grassi M., Di Cagno M.P., Tho I. (2025). A simplified method to interpret the mechanism of drug release from thin polymeric films by drug diffusivity measurements. Int. J. Pharm..

[82] Cui S., Bahraminia M., Rouabhia M., Semlali A., Béland F., Zhang Z. (2024). Design, characterization, and release profile of a cannabidiol (CBD)-rich polyvinyl alcohol hydrogel. Mater. Adv..

[83] Zhang H., Lan L.-M., Hu H.-J., Chen Y., Hu T., Cheng H., Hu X., Tang S., Liao X.-P., Jiang G.-B. (2025). Cannabidiol-loaded hydrogel contact lenses for on-demand pH regulation and enhanced corneal alkali burn repair. J. Control. Release.

[84] Wang C., Cui B., Sun Y., Wang C., Guo M. (2022). Preparation, stability, antioxidative property and in vitro release of cannabidiol (CBD) in zein-whey protein composite nanoparticles. LWT.

[85] Sharkawy A., Barreiro F., Rodrigues A. (2022). Pickering emulsions stabilized with chitosan/gum Arabic particles: Effect of chitosan degree of deacetylation on the physicochemical properties and cannabidiol (CBD) topical delivery. J. Mol. Liq..

[86] Lozza I., Martín-Sabroso C., Hurtado-Marcos C., Montejo-Rubio C., Fraguas-Sánchez A.I., Torres-Suárez A.I. (2025). Cannabidiol-loaded-injectable depot formulation for the treatment of triple-negative breast cancer: Design, development, in-vitro and in-ovo evaluation of its anticancer activity. Int. J. Pharm..

[87] Wang C., Li J., Sun Y., Wang C., Guo M. (2022). Fabrication and characterization of a cannabidiol-loaded emulsion stabilized by a whey protein-maltodextrin conjugate and rosmarinic acid complex. J. Dairy Sci..

